# Supplementary material for: Aberrant GlyRS-HDAC6 interaction linked to axonal transport deficits in Charcot-Marie-Tooth neuropathy
Source: Nat Commun. 2018 Mar 8;9:1007. doi: 10.1038/s41467-018-03461-z (PMC5843656; doi:10.1038/s41467-018-03461-z)
Supplement: Supplementary file 1 — Supplementary Information [file 41467_2018_3461_MOESM1_ESM.pdf]

SUPPLEMENTARY INFORMATION

Aberrant GlyRS-HDAC6 interaction links to axonal transport deficit in Charcot-Marie-Tooth neuropathy

Mo *et.al.*

Supplementary Figure 1. Aberrant HDAC6 interaction is specifically induced by GlyRS mutations identified in CMT2D patients.

Supplementary Figure 2. $GlyRS^{P234KY}$ binds to HDAC6 catalytic domains.

Supplementary Figure 3. Quantification of $GlyRS^{CMT2D}$ interaction with HDAC6.

Supplementary Figure 4. CMT2D mice exhibit decreased levels of acetylated α-tubulin in sciatic nerves.

Supplementary Figure 5. Representative images of the QD-NGF axonal transport assay.

Supplementary Figure 6. Uncropped images of Western blots.

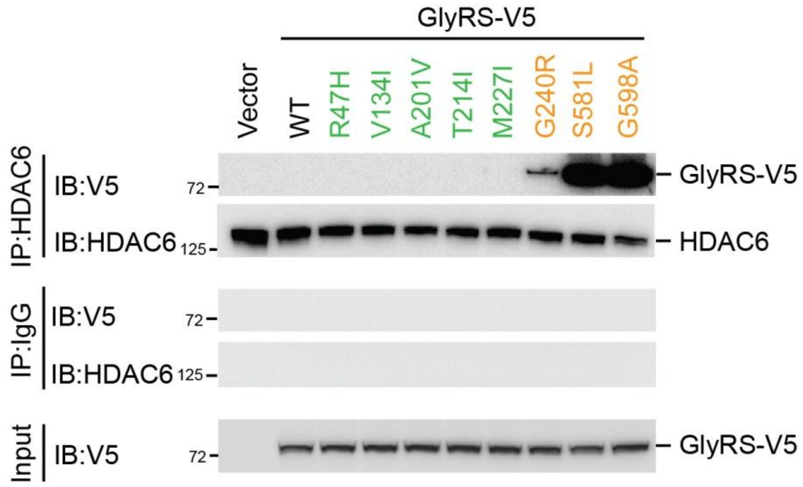

Supplementary Figure 1. Aberrant HDAC6 interaction is specifically induced by GlyRS mutations identified in CMT2D patients. Co-immunoprecipitation using transfected NSC-34 cells showing that GlyRS variants identified in the general population (green) do not induce the aberrant GlyRS-HDAC6 interaction, in contrast to mutations associated with CMT patients (orange), which do interact with HDAC6.

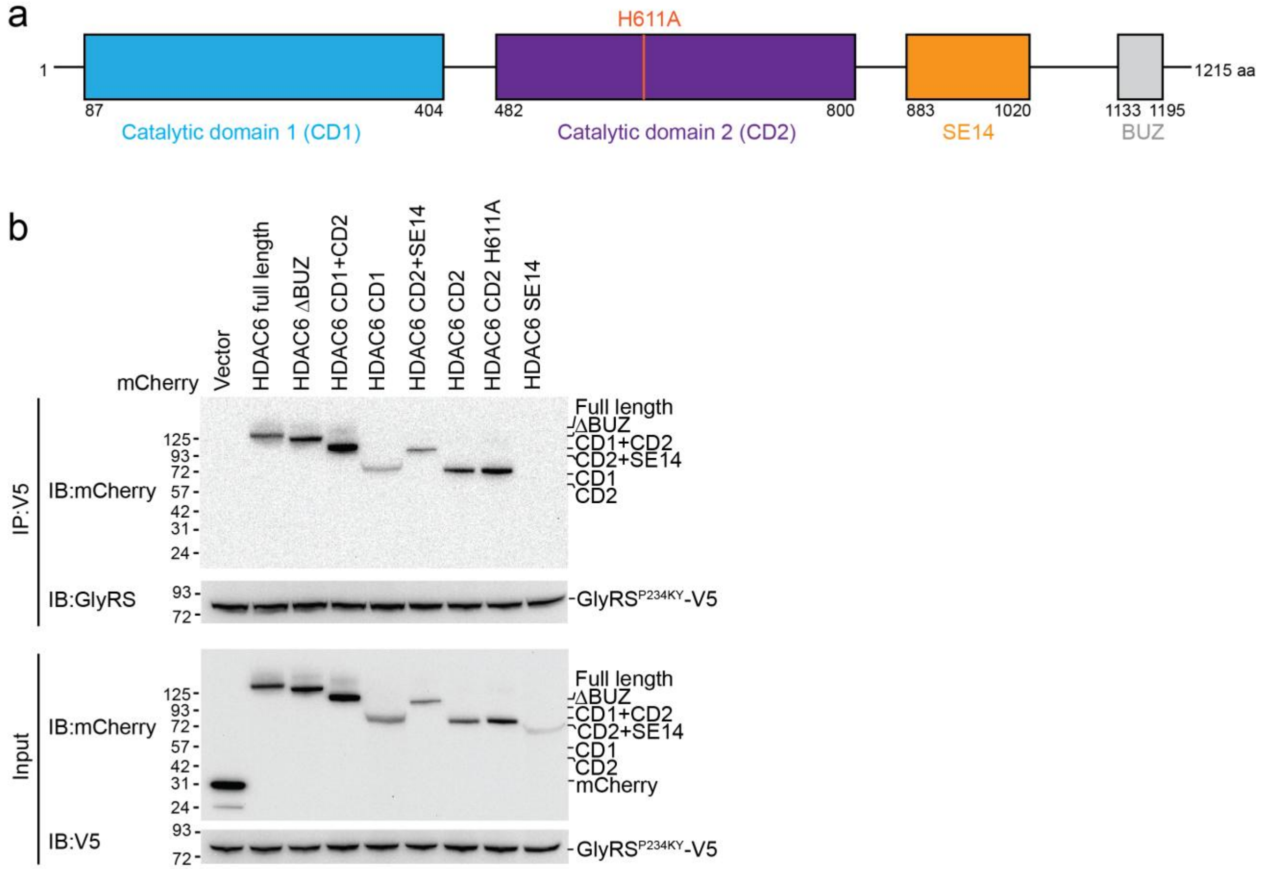

Supplementary Figure 2. GlyRS^{P234KY} binds to HDAC6 catalytic domains. (**a**) Domain composition of human HDAC6. H611A is an active site mutation that abolishes the deacetylase activity of HDAC6. (**b**) Co-immunoprecipitation using transfected HEK293 cells to map the binding site of GlyRS^{P234KY} on HDAC6. GlyRS^{P234KY} is c-terminal V5 tagged, while HDAC6 (full length and fragments) are mCherry tagged on the N-terminus.

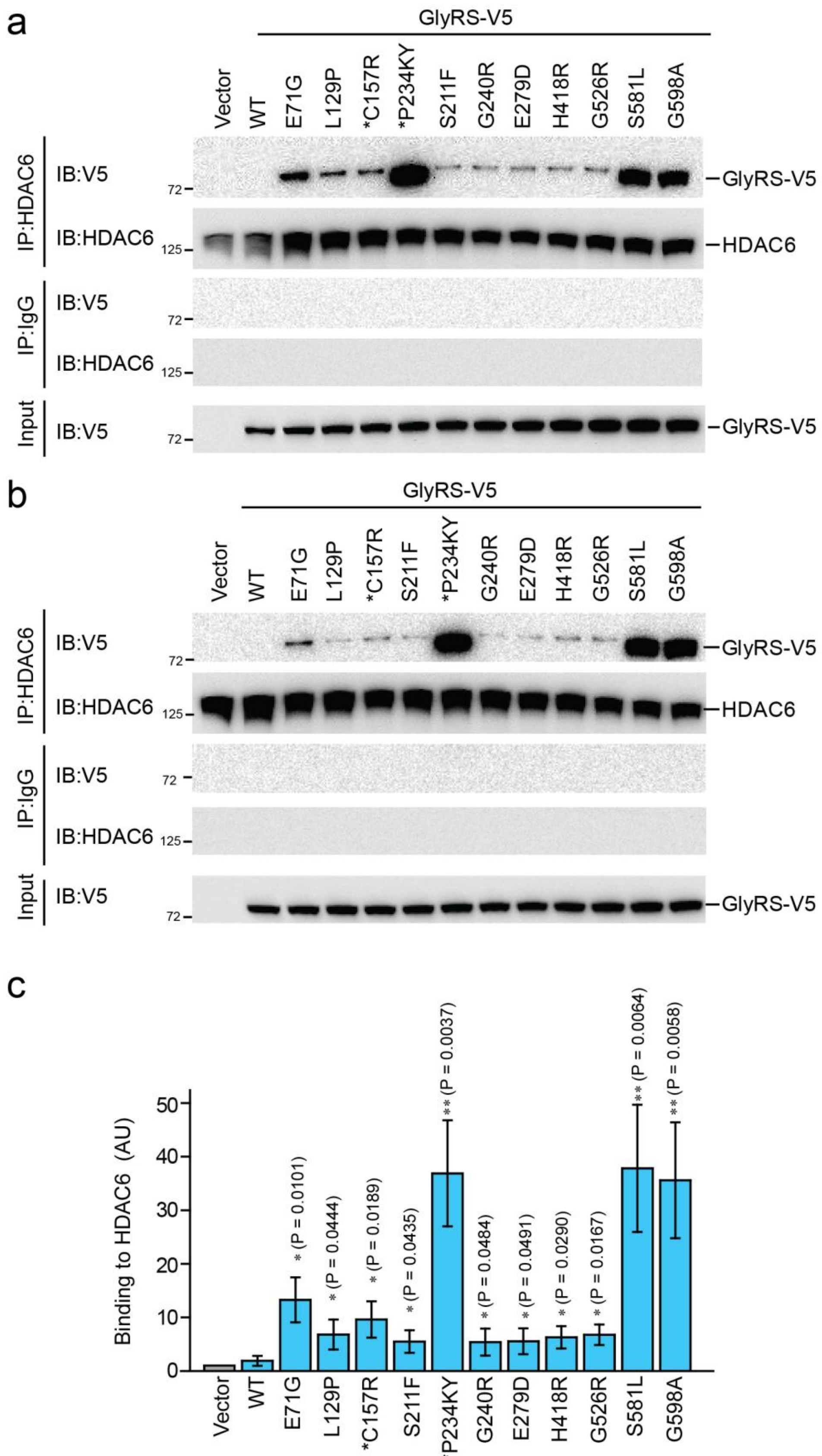
a
GlyRS-V5
Vector
WT
E71G
L129P
*C157R
*P234KY
S211F
G240R
E279D
H418R
G526R
S581L
G598A
IP:HDAC6
IB:V5
IB:HDAC6
IP:IgG
IB:V5
IB:HDAC6
Input
IB:V5
72
125
GlyRS-V5
HDAC6
b
GlyRS-V5
Vector
WT
E71G
L129P
*C157R
S211F
*P234KY
G240R
E279D
H418R
G526R
S581L
G598A
IP:HDAC6
IB:V5
IB:HDAC6
IP:IgG
IB:V5
IB:HDAC6
Input
IB:V5
GlyRS-V5
HDAC6
c
Binding to HDAC6 (AU)
0
10
20
30
40
50
* (P = 0.0101)
* (P = 0.0444)
* (P = 0.0189)
* (P = 0.0435)
** (P = 0.0037)
* (P = 0.0484)
* (P = 0.0491)
* (P = 0.0290)
* (P = 0.0167)
** (P = 0.0064)
** (P = 0.0058)
Vector
WT
E71G
L129P
*C157R
S211F
*P234KY
G240R
E279D
H418R
G526R
S581L
G598A

Supplementary Figure 3. Quantification of GlyRS^{CMT2D} interaction with HDAC6. (**a, b**) Two replicates of co-immunoprecipitation showing that GlyRS^{CMT2D} proteins (C-terminal V5-tagged), but not GlyRS^{WT}, can bind to HDAC6 **(**endogenous) in transfected NSC-34 cells. (**c**) Quantification of the co-immunoprecipitation experiments showing that, despite variability in strength, all GlyRS^{CMT2D} mutants tested aberrantly interact with HDAC6. The amounts of HDAC6-bound GlyRS were quantified with ImageJ and normalized against the values of vector control. Statistical analysis was done with two-tailed unpaired student's t-test. n = 3 biological replicates. Data are presented as means ± s.d.

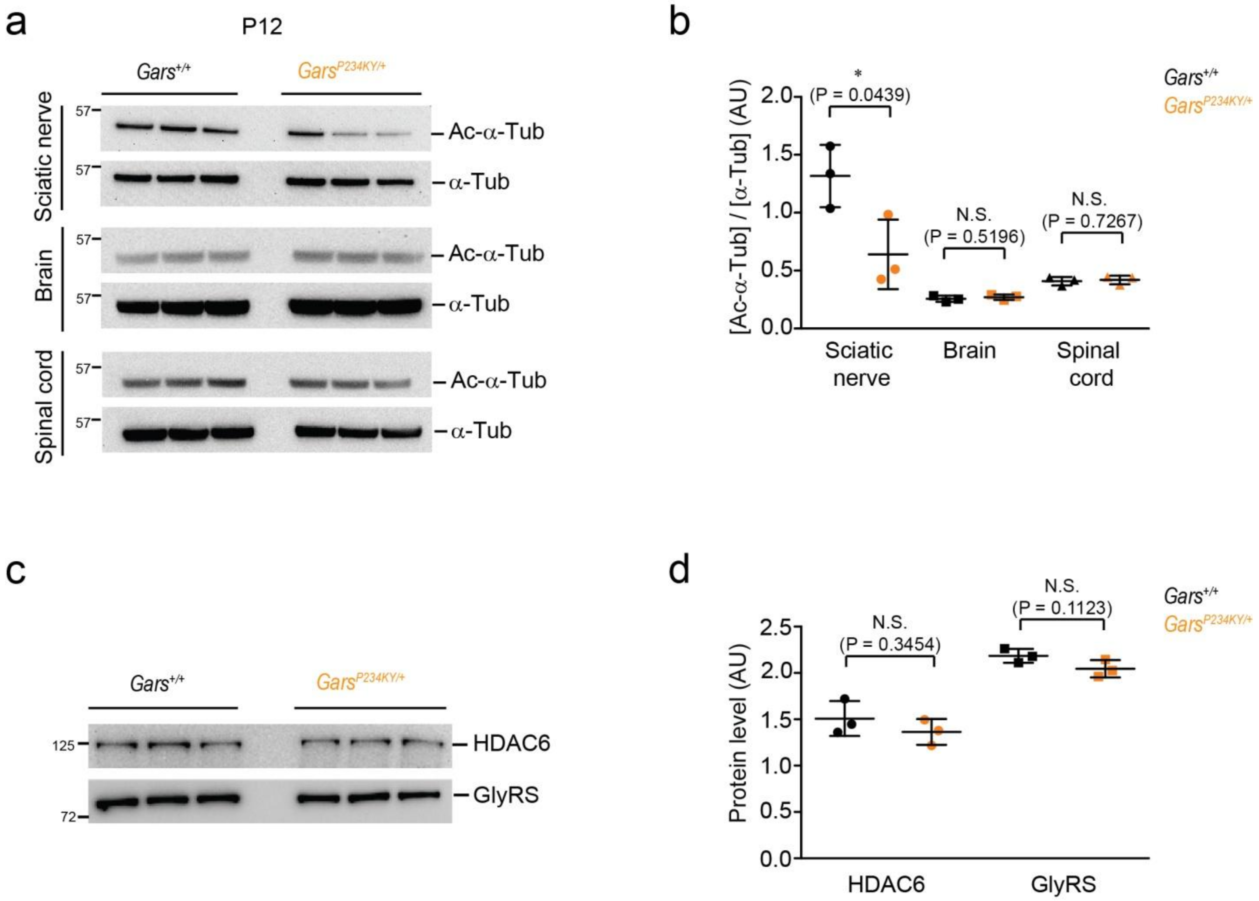

Supplementary Figure 4. CMT2D mice exhibit decreased levels of acetylated α-tubulin in sciatic nerves. (**a, b**) Western blot analysis (**a**) and quantification (**b**) showing decreased α-tubulin acetylation in sciatic nerves of CMT2D mice. Postnatal day 12 *Gars*$^{+/+}$ and *Gars*$^{P234KY/+}$ littermates were used for the analysis. (**c, d**) Western blot analysis (**c**) and quantification (**d**) showing no significant difference in HDAC6 and GlyRS levels in sciatic nerves of *Gars*$^{+/+}$ and *Gars*$^{P234KY/+}$ littermates (P12). The protein levels were quantified with ImageJ. Statistical analyses were done with two-tailed unpaired student's t-test. Data are presented as means ± s.d. n=3 mice per group.

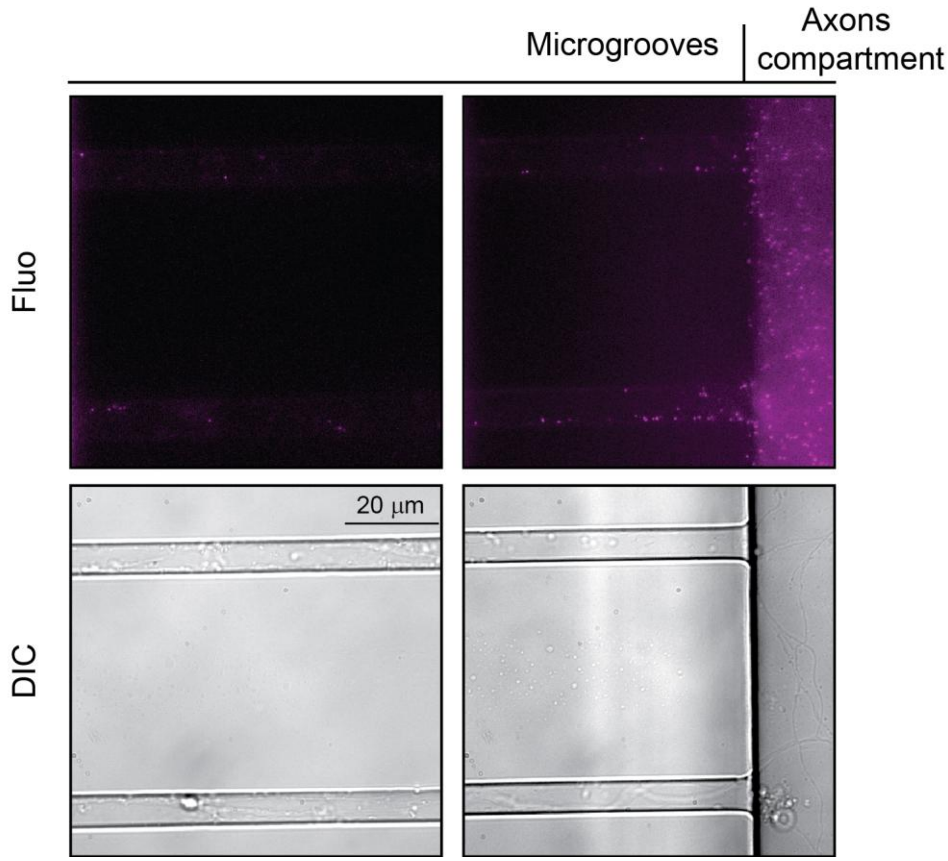

Supplementary Figure 5. Representative images of the QD-NGF axonal transport assay. Fluorescence and differential interference contrast (DIC) images of microgrooves and the axon compartment. No QD signal was observed in the microgrooves without axons.

Figure 1b

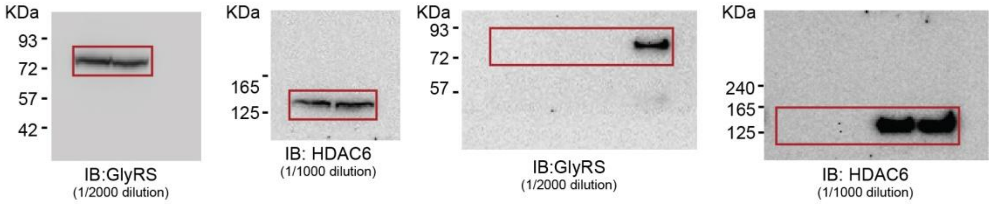

Figure 1c

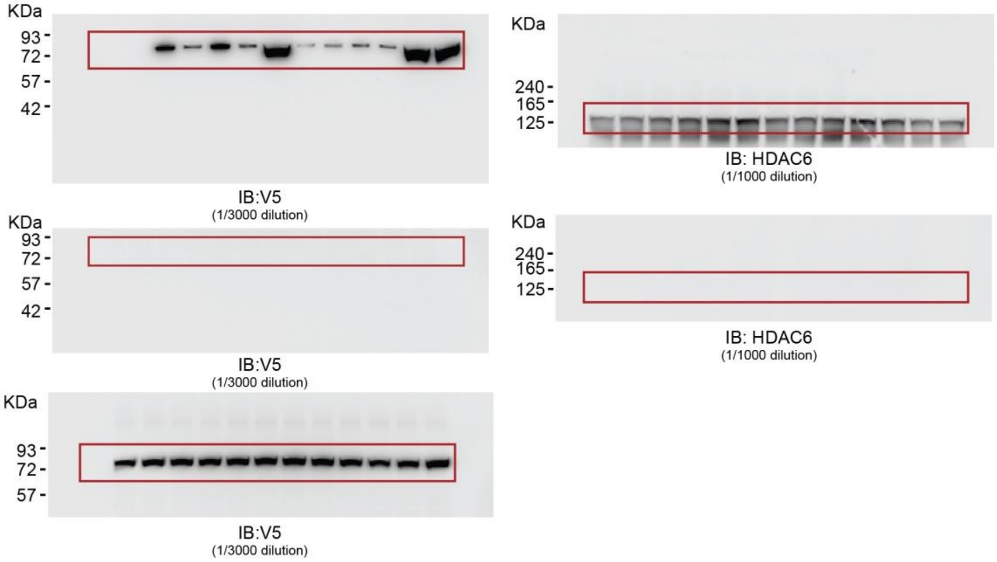

Figure 1d

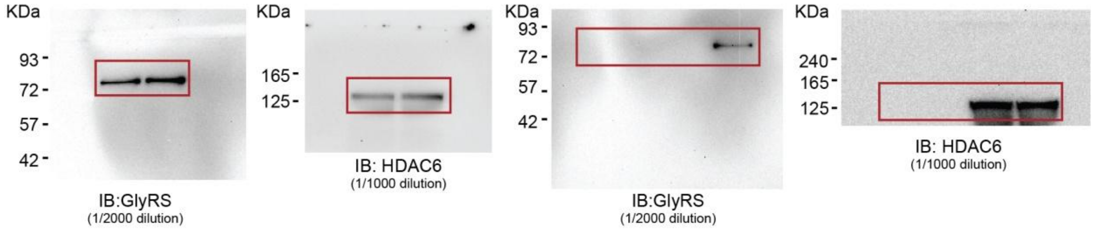

Figure 1f

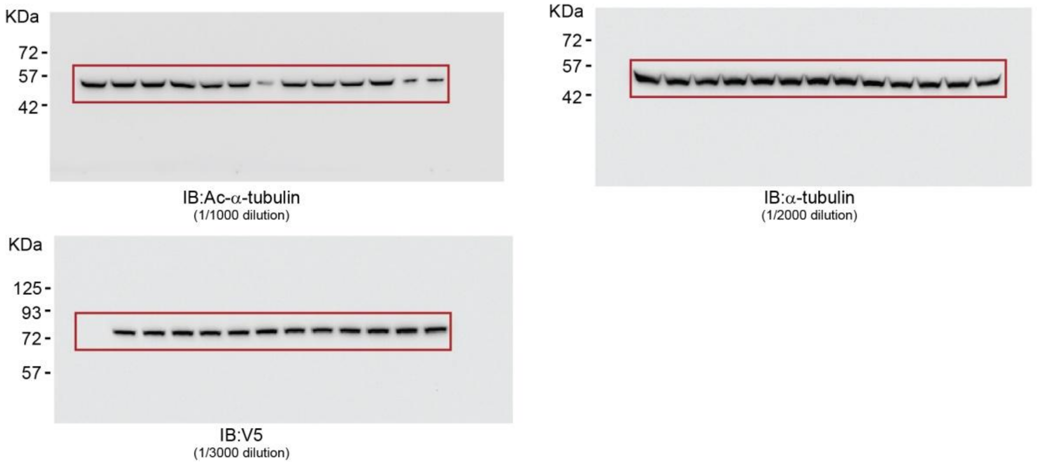

Figure 2a

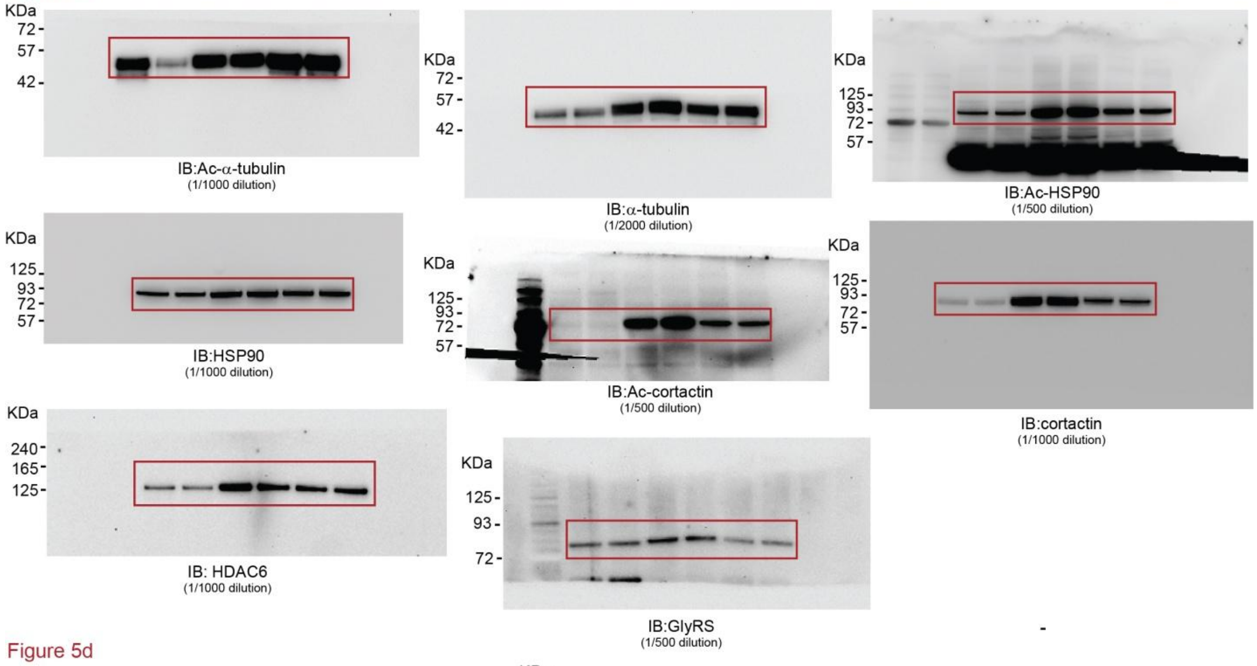

Figure 5d

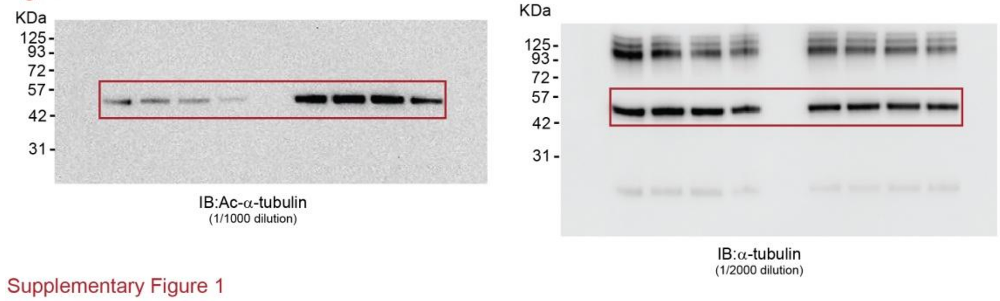

Supplementary Figure 1

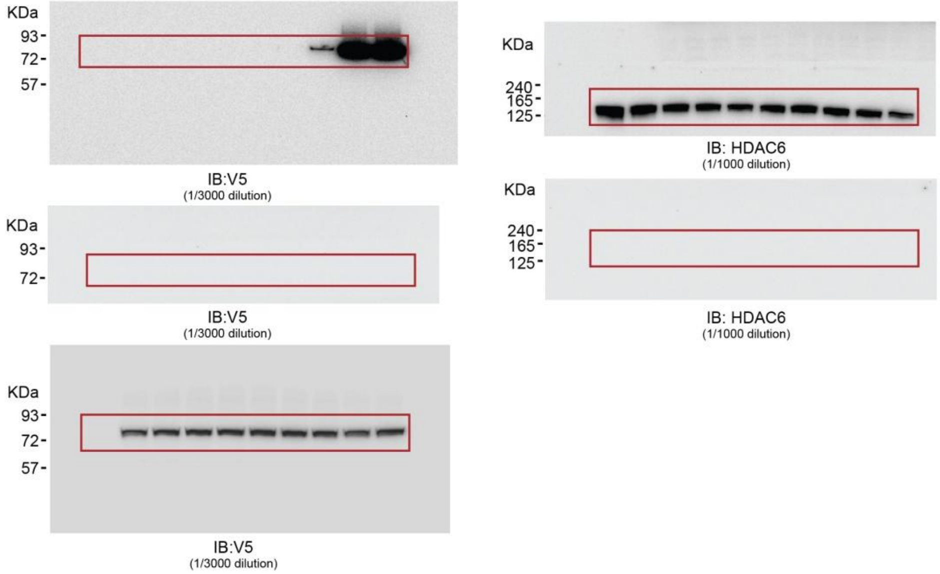

Supplementary Figure 2

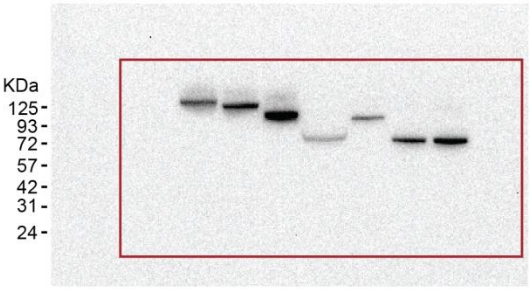

IB: mCherry
(1/500 dilution)

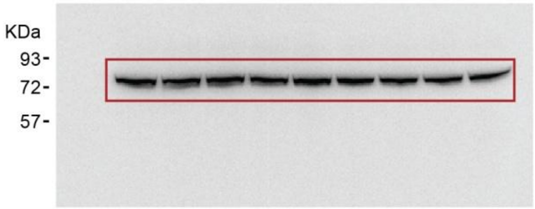

IB:GlyRS
(1/500 dilution)

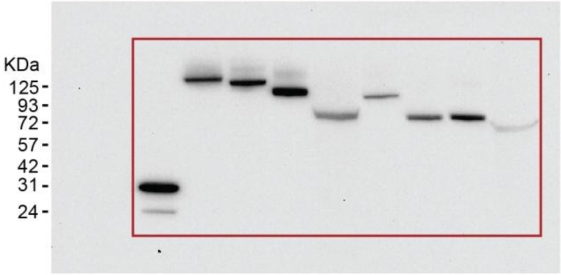

IB: mCherry
(1/500 dilution)

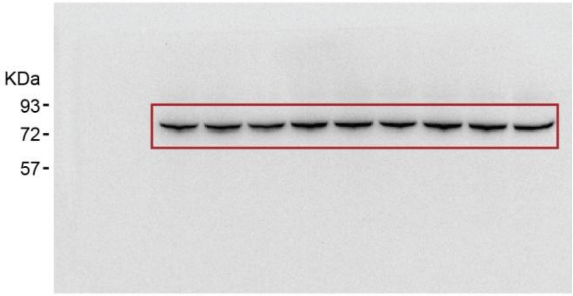

IB:V5
(1/3000 dilution)

Supplementary Figure 3a

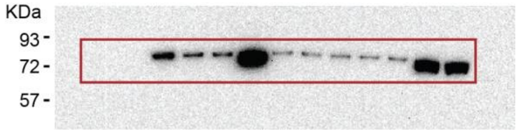

IB:V5
(1/3000 dilution)

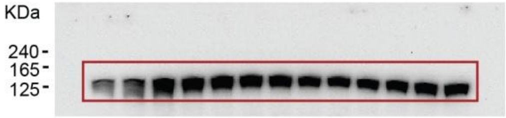

IB: HDAC6
(1/1000 dilution)

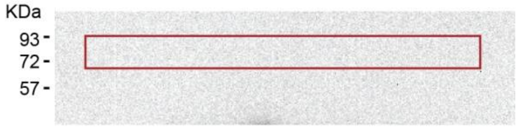

IB:V5
(1/3000 dilution)

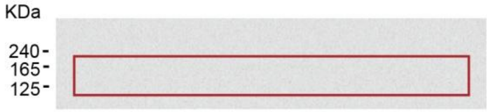

IB: HDAC6
(1/1000 dilution)

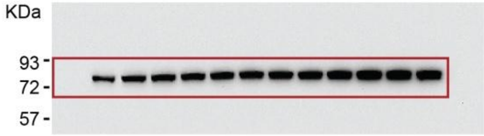

IB:V5
(1/3000 dilution)

Supplementary Figure 3b

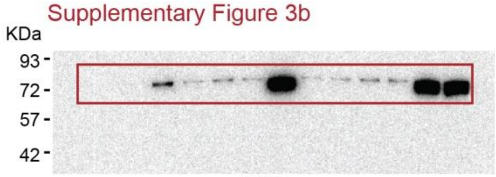

IB:V5
(1/3000 dilution)

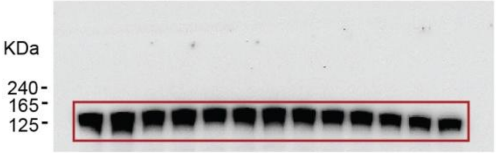

IB: HDAC6
(1/1000 dilution)

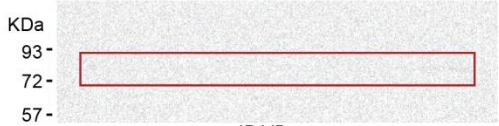

IB:V5
(1/3000 dilution)

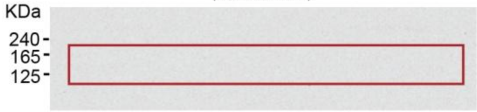

IB: HDAC6
(1/1000 dilution)

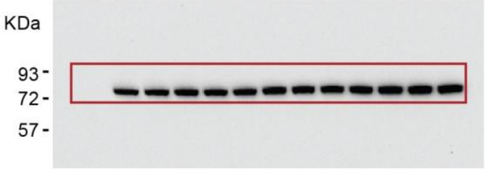

IB:V5
(1/3000 dilution)

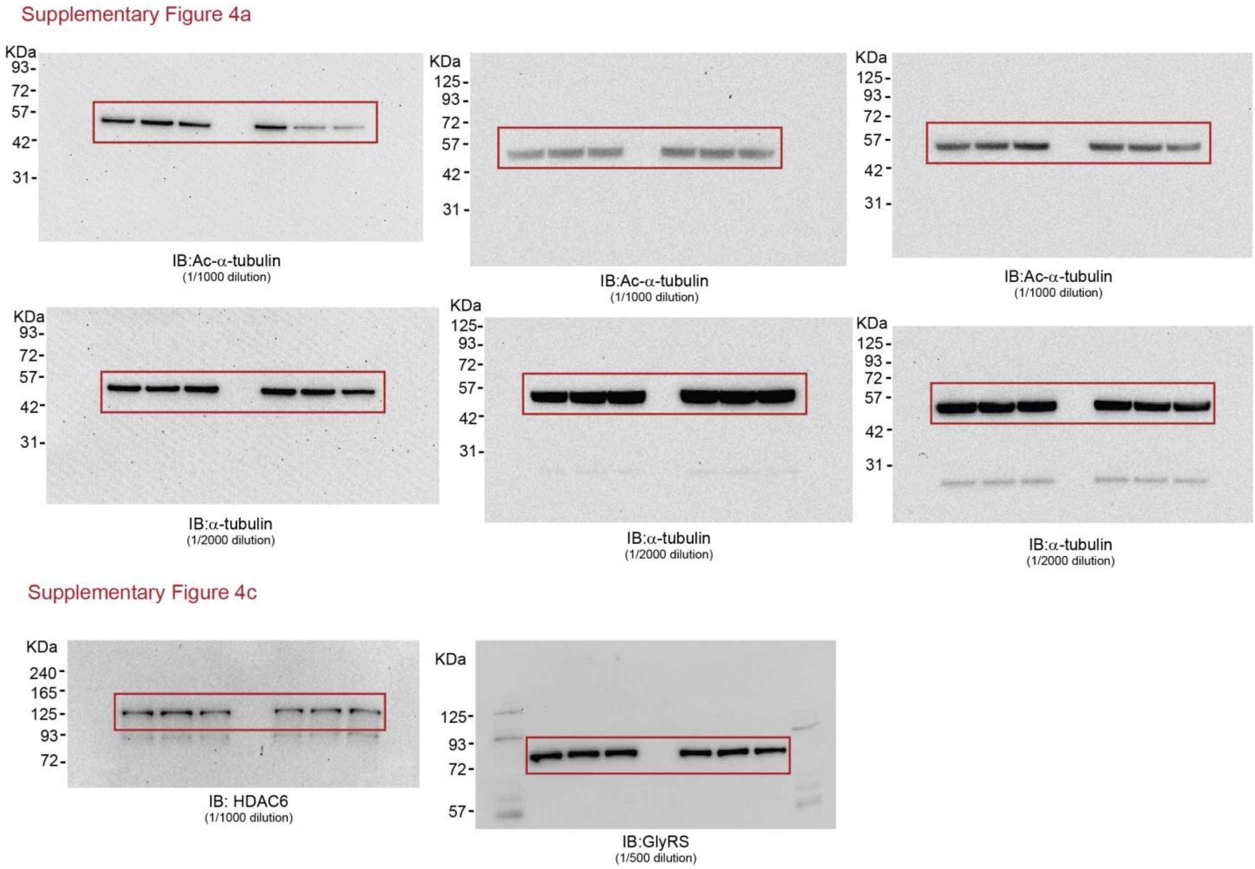

Supplementary Figure 6. **Uncropped images of Western blots.** Dilutions of antibodies are as indicated.
