# Supplementary material for: Aberrant GlyRS-HDAC6 interaction linked to axonal transport deficits in Charcot-Marie-Tooth neuropathy
Source: Nat Commun. 2018 Mar 8;9:1007. doi: 10.1038/s41467-018-03461-z (PMC5843656; doi:10.1038/s41467-018-03461-z)
Supplement: Supplementary file 3 — Description of Additional Supplementary Files [file 41467_2018_3461_MOESM3_ESM.pdf]

File Name: Supplementary Software 1
Description: ImageJ macro to generate kymographs for axonal transport analysis.

File Name: Supplementary Movie 1
Description: QD-NGF movement in DRG axons of postnatal day 12 *Gars*$^{+/+}$ mice.

File Name: Supplementary Movie 2
Description: QD-NGF movement in DRG axons of postnatal day 12 *Gars*$^{P234KY/+}$ mice.

File Name: Supplementary Movie 3
Description: QD-NGF movement in DRG axons of postnatal day 12 *Gars*$^{+/+}$ mice in response to solvent control.

File Name: Supplementary Movie 4
Description: QD-NGF movement in DRG axons of postnatal day 12 *Gars*$^{+/+}$ mice in response to Tub A.

File Name: Supplementary Movie 5
Description: QD-NGF movement in DRG axons of postnatal day 12 *Gars*$^{P234KY/+}$ mice in response to solvent control.

File Name: Supplementary Movie 6
Description: QD-NGF movement in DRG axons of postnatal day 12 *Gars*$^{P234KY/+}$ mice in response to Tub A.
